# The stories about racism and health: the development of a framework for racism narratives in medical literature using a computational grounded theory approach

**DOI:** 10.1186/s12939-023-02077-0

**Published:** 2023-12-21

**Authors:** Caroline A. Figueroa, Erin Manalo-Pedro, Swetha Pola, Sajia Darwish, Pratik Sachdeva, Christian Guerrero, Claudia von Vacano, Maithili Jha, Fernando De Maio, Chris J. Kennedy

**Affiliations:** 1 University of California Berkeley, Berkeley, USA; 2 Department of Technology, Policy, and Management, Delft University of Technology, Policy & Management Room B3.230, Building 31, Jaffalaan 5, Delft, 2628 BX the Netherlands; 3 Fielding School of Public Health, University of California Los Angeles, Los Angeles, USA; 4 School of Public Health, University of California Berkeley, Berkeley, USA; 5 American Medical Association, Chicago, USA; 6 Department of Sociology, DePaul University, Chicago, USA; 7 Department of Psychiatry, Harvard Medical School, Boston, USA; 8 Center for Precision Psychiatry, Massachusetts General Hospital, Boston, USA

**Keywords:** Health Equity, Racism, Social Justice, Medicine, Narratives, Computational grounded theory, Medical journals

## Abstract

**Introduction:**

The scientific study of racism as a root cause of health inequities has been hampered by the policies and practices of medical journals. Monitoring the discourse around racism and health inequities (i.e., racism narratives) in scientific publications is a critical aspect of understanding, confronting, and ultimately dismantling racism in medicine. A conceptual framework and multi-level construct is needed to evaluate the changes in the prevalence and composition of racism over time and across journals.

**Objective:**

To develop a framework for classifying racism narratives in scientific medical journals.

**Methods:**

We constructed an initial set of racism narratives based on an exploratory literature search. Using a computational grounded theory approach, we analyzed a targeted sample of 31 articles in four top medical journals which mentioned the word ‘racism’. We compiled and evaluated 80 excerpts of text that illustrate racism narratives. Two coders grouped and ordered the excerpts, iteratively revising and refining racism narratives.

**Results:**

We developed a qualitative framework of racism narratives, ordered on an anti-racism spectrum from impeding anti-racism to strong anti-racism, consisting of 4 broad categories and 12 granular modalities for classifying racism narratives. The broad narratives were “dismissal,” “person-level,” “societal,” and “actionable.” Granular modalities further specified how race-related health differences were related to racism (e.g., natural, aberrant, or structurally modifiable). We curated a “reference set” of example sentences to empirically ground each label.

**Conclusion:**

We demonstrated racism narratives of dismissal, person-level, societal, and actionable explanations within influential medical articles. Our framework can help clinicians, researchers, and educators gain insight into which narratives have been used to describe the causes of racial and ethnic health inequities, and to evaluate medical literature more critically. This work is a first step towards monitoring racism narratives over time, which can more clearly expose the limits of how the medical community has come to understand the root causes of health inequities. This is a fundamental aspect of medicine’s long-term trajectory towards racial justice and health equity.

**Supplementary Information:**

The online version contains supplementary material available at 10.1186/s12939-023-02077-0.

## Introduction

Differences in health outcomes by race, often referred to as*“racial health inequities,”* are rooted in racism [1–4]. Racial health inequities are global and measurable [5, 6]. For example, in the US, state-level structural racism (social, economic, and policy context) shows associations with higher infant mortality, myocardial infarction, depression, Body Mass Index, and worse self-rated health among Black people [5]. Yet, few empirical studies that explicitly mention racism have been published in leading medical journals [7].

ifferences in morbidity and mortality have often been viewed by scientists through an individual-focused lens rooted in beliefs, behaviors, or biology. This became apparent during the COVID-19 pandemic, where higher mortality for Black and Hispanic/Latinx communities in the US was initially explained, in many publications and venues, through mechanisms such as genetics, risk tolerance, and hygiene, without substantial evidence [8]. Recurring patterns of how racial health inequities are framed generate *racism narratives* [9]. Understanding and changing harmful narratives is a fundamental component to medicine’s long-term trajectory towards racial justice and health equity [10].

Narratives that marginalize the effects of racism on health inequities can have wide-reaching consequences across the healthcare ecosystem. The situation leads to several concerning consequences. Racism in health is not adequately recognized as valid scientific topics, causing an oversight of the detrimental effects of racism among the scientific community, funding entities, and the wider public. Furthermore, researchers who emphasize racism and health are often sidelined from well-regarded journals. This exclusion can hinder their career progression and chances of securing funding. Moreover, this environment fosters the formulation of policies that either perpetuate or exacerbate existing health disparities [7]. Alternatively, anti-racist narratives (1) acknowledge that the unequal distribution of disease across racial groups is rooted in legacies of racial hierarchies, (2) illuminate contemporary racist mechanisms, and (3) promote actions to dismantle the structures that maintain inequity [11]. Thus, providing an approach to studying these narratives is essential for shedding light on the mechanisms perpetuating health inequities.

However, the scientific study of racism narratives that exist in peer-reviewed medical literature is still in its infancy. Medical literature plays a crucial role in disseminating the latest research findings, clinical guidelines, and best practices to the medical and healthcare communities, en thereby has a high impact on clinical medicine.The American Medical Association’s 2021-23 Strategic Plan to Embed Racial Justice and Advance Health Equity calls for an increase in the frequency and visibility of research studies that disrupt dominant narratives on racism and health, and expose harm from racism [12] some work has examined the inclusion of the word “racism” in influential medical journals, tracking trends over time and contrasting the use of the word in empirical versus commentary papers. Further, narratives in medicine have been assessed via discourse analysis of clinicians’ perspectives on health care disparities.8 However, approaches to examining narratives in peer-reviewed medical articles specifically are lacking.

Understanding what narratives are present in published research, especially in prestigious and widely-cited medical journals, is key to evaluating how knowledge production in the medical field on racial health inequities perpetuates, or dismantles, racism.

Our research aims to fill this important gap by providing a comprehensive and data-driven approach to studying racism narratives, essential for shedding light on the mechanisms perpetuating health inequities. Leveraging techniques developed in computational grounded theory and measurement theory [13–16] we aim to provide a thorough assessment of the discourse around racism as a root cause of health inequities in leading medical journals. The objective of this study was to develop a framework of racism narratives by categorizing and ordering narratives with excerpts from influential medical studies. This study is a first step in providing a methodological process to monitor the stories the medical literature tells about racism and health. Ultimately, this methodology can form an important component of ongoing efforts to reduce harmful dominant narratives in medical knowledge and practices.

## Methods

We developed a conceptual framework for describing racism narratives in medicine by employing an iterative, theory-driven, and data-informed approach. This conceptual framework aligns closely with the notion of a *construct* from measurement theory [17]. The construct of racism narratives consists of several levels along a spectrum between two extremes. Based on an exploratory literature review (see Supplementary Material for details, and Table S1 for an overview of the included articles), we conceptualized these two extremes as “dismissal of racism” to “actionable anti-racism.” We then developed a reference set of excerpts from influential medical journals to represent levels along the spectrum. Lastly, we clustered similar excerpts into labeled groups of racism narratives.

### Sampling strategy

For this analysis we chose a corpus of articles derived from a previous search by Krieger et al. in March 2021 [18]. The study focused on the term “racism” because acknowledging and addressing racism is a critical step in understanding its impact on healthcare. The articles included the word “racism” and were published between January 1, 1990 and December 31, 2020 by four leading medical journals (with impact factors > 30 for 2019). The researchers obtained and reviewed PDFs of the identified papers and categorized them into two groups: (a) empirical studies or review papers with significant data components, and (b) non-empirical or non-data-driven papers.

We selected articles from high-impact journals that influence the medical field: the *New England Journal of Medicine* (NEJM), *The Lancet*, the *Journal of the American Medical Association* (JAMA), and the *British Medical Journal* (BMJ). Each article mentioned the word “racism” anywhere in the article at least once [19].

We chose to focus on the leading medical journals, because they have a significant influence on the practice of medicine. They play a crucial role in disseminating the latest research findings, clinical guidelines, and best practices to the medical and healthcare communities. We selected articles from 1960 to 2020, because we wanted to understand narratives that have historically shaped the field of medicine’s view of health inequities and racism.

### Developing a reference set

We developed a “reference set” to illustrate levels of anti-racism narratives. A reference set plays a role in providing concrete examples or manifestations of the underlying construct. It serves as a touchstone for calibrating and interpreting a theoretical construct, aiding in the validation and refinement of the construct, which describes a progression of the construct from lower to higher levels. The reference set is vital to ensure that the model accurately represents the complexity and multifaceted nature of the construct. Constructs represent phenomena that are not directly observable but can be inferred in observed data.

Articles were iteratively selected across journals, decades, and article types until the authors felt each level of anti-racism was represented. Four coders (CAF, EMP, CK, and SP) read full text articles to examine how “racism” was mentioned. Each coder gathered example sentences from these articles that either contained the term “racism” or alluded to racial health inequities without the term “racism” (the article needed to have the word ‘racism’ in the body of the text, but not all excerpts contained the word racism).

### Labeling levels of anti-racism

Two coders (CAF and EMP) independently grouped similar excerpts into clusters of racism narratives using separate Padlets (https://padlet.com/, a platform for online collaboration). Our conceptualizations of narratives around racism and health considered how explicitly racism was named, how racism was described in relation to health (e.g., as a product of socioeconomic status, physician bias, medical mistrust, and individual behaviors), and whether actions against racism were mentioned in these excerpts.

We conducted two rounds of coding. After each round, we compared our results, discussed coding decisions, and refined our labels. The first round produced a framework of broad and granular labels of anti-racism narratives. During the second round, we reassessed 10 excerpts (12.5%) that appeared to be incongruent.

#### Researchers’ characteristics

Our diverse backgrounds (e.g. identifying as Latinx, Asian, immigrat and female) influenced the research questions, approach to developing this framework, and interpretation of results. Our multidisciplinary team leveraged expertise within and beyond healthcare, including data science, medicine, public health, and education leading to diverse views on the topic. Our varied perspectives on equity have been shaped through formal study of and direct experiences with racism.

#### Ethics

Ethical consent was not necessary as we did not use human subjects data.

## Results

Our reference set contained 80 excerpts from 31 articles published in *The Lancet* (10), *BMJ* (9), *JAMA* (7), and *NEJM* (5). The articles were published between 1960 and 2020, with the majority after 2010. Table 1 summarizes the journals, decades, and types of the articles. Supplemental Tables 2 and 3 show excerpts clustered by journal and year respectively.

**Table 1:** Coded excerpts by journal and year of publication

	Timeframe							
	1969–1999		2000–2009		2010–2020		Grand Total	
Journal	n	row %	n	row %	n	row %	n	col %
NEJM	0	0.0%	16	57.1%	12	42.9%	28	35.0%
JAMA	2	10.5%	3	15.8%	14	73.7%	19	23.8%
The Lancet	5	26.3%	0	0.0%	14	73.7%	19	23.8%
BMJ	5	35.7%	7	50.0%	2	14.3%	14	17.5%
Grand Total	12		26		42		80	

**Fig. 1:**
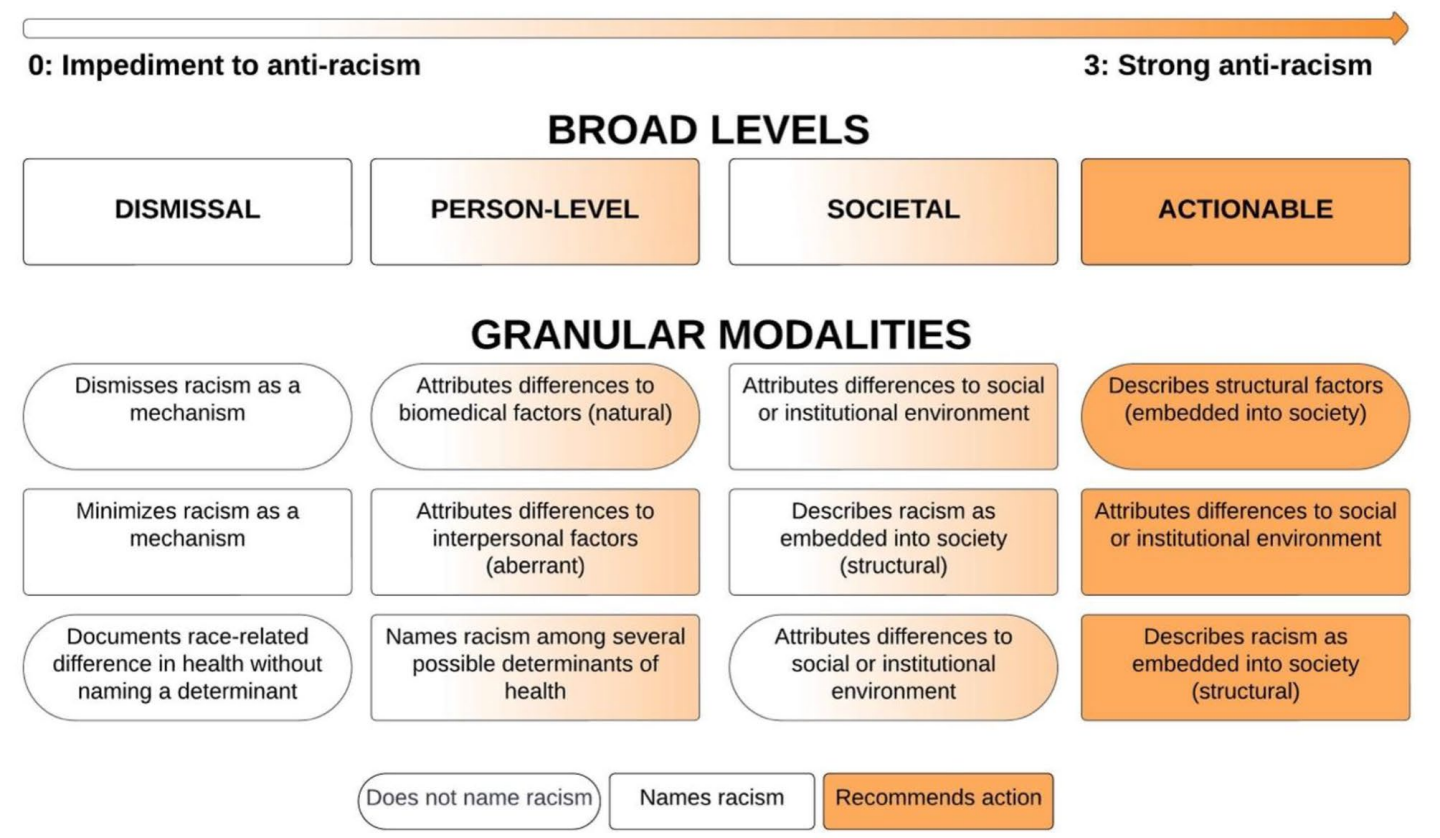
Theorization of anti-racism narratives with broad levels and granular modalities

We ordered narrative labels on a spectrum of anti-racism. We theorized four broad levels of anti-racism narratives: dismissal, person-level, societal, and actionable. We additionally theorized 12 granular modalities to capture nuance in the narratives. The low extreme (impediment to anti-racism) was “Dismisses racism as a mechanism; does not name racism” while the high extreme (strong anti-racism) was “Describes racism as structural (embedded into society); recommends action.” Fig. 1 illustrates our theorization of anti-racism narratives with broad levels and granular modalities.

### Broad levels

We theorized four broad levels of anti-racism narratives: dismissal, person-level, societal, and actionable. We provide definitions of each level in Table 2.

**Table 2:** Definitions of broad narratives

**Dismissal**	Authors dismiss the role of racism and/or provide none or alternative explanations for racial/ethnic health inequities. The alternative explanations are related to biological/genetic differences or focus solely on behavior/lifestyle (blaming the victim).
**Person-level**	Authors discuss racial/ethnic health inequities and provide interpersonal explanations like interpersonal racism, behavior, physician bias or income/education (without mentioning social disadvantage). Authors may or may not mention the word racism in the excerpt. They provide no anti-racism strategies.
**Societal**	Authors discuss racial/ethnic health inequities and provide social/institutional explanations beyond biology, genetics, behavior, or physician bias (e.g., historical injustices, social inequalities, access). Authors may or may not mention the word racism and provide limited or no actionable strategies at the structural level.

We defined “dismissal” narratives as those that denied the existence of racism in society or who sought race-evasive (sometimes called “colorblind”–itself now interpreted by many as ableist) solutions. The first row in Table 3 includes an example of a dismissal narrative from a 1976 commentary in the NEJM [20]. Although the author acknowledges that “racial discrimination … remains in American medicine,” they suggest that such discrimination could be eliminated by selecting decision makers for boards and committees “without regard to criteria like race, creed or color.” By invoking the narrative that “all physicians of goodwill” are doing their best, the excerpt describes a race-evasive approach that dismisses the lack of representation within positions of power as perpetuating inequities, while assuming that racial representation precludes having the most qualified provide leadership In other words, it is assumed that people of color are inherently less qualified.

**Table 3:** Example excerpts with broad and granular labels

Broad level	Granular modality	Example excerpt
**Dismissal**	Dismisses racism as a mechanism; does not name racism	*In a time when all physicians of goodwill are working to eliminate the racial discrimination that remains in American medicine, it is most disheartening to read Dr. Therman E. Evans’s call for prescription of racial quotas on all “advisory panels, review committees and policy boards” (N Engl J Med 295:1013, 1976). That the men and women who serve on such boards and committees should be selected for their service on merit, and without regard to criteria like race, creed or color, should not need to be stated.* [20]
**Person-level**	Attributes race-related difference in health to interpersonal (aberrant) factors	*Higher levels of implicit bias among clinicians have been directly linked with biased treatment recommendations in the care of black patients, although the pattern is not uniform. *[21]
**Societal**	Describes structural (embedded into society) factors; does not name racism	*Our results provide key evidence that the plurality of HIV-related disparities in US black MSM relative to other MSM are disparities in HIV clinical care access and use, structural issues (e.g., low income, unemployment, incarceration, low education), and sex partner characteristics, rather than disparities in sexual and substance-use risk behaviours. Low income, unemployment, incarceration, and low education are not only interrelated, but also all are independently associated with HIV infection … Possible intervention strategies for black MSM overall, HIV-positive black MSM, and young black MSM to address these and other disparities are outlined in the appendix.* [22]
**Actionable**	Describes racism as structural (embedded into society); recommends action	*Although there is much to do, we recommend that health care systems engage, at the very least, in five practices to dismantle structural racism and improve the health and well-being of the black community and the country.* [23]

We considered “person-level” narratives as explanations for differences in health by race or ethnicity that were framed at the individual and/or interpersonal level. These included interpersonal racism, behavior, or physician bias. If socioeconomic status was mentioned, it was framed as an individual attribute, such as income or education, without the social context of disadvantage. The person-level excerpt in Table 3 focuses on individual level clinician factors as a driving factor between health inequities. Of note, though the focus of the commentary is on clinician bias, and the need for increasing awareness, the authors mention systemic factors, stating that racial biases influence institutional factors and advocating for multilevel policies to increase access to healthy living. Within “societal” narratives, authors framed institutions, society, or systems as determinants of racial/ethnic health inequities. Strategies for dismantling racism are not explicitly mentioned. The societal level excerpt (Table 3) from a 2012 empirical paper in The Lancet emphasized “structural issues” as contributing to HIV-related disparities among Black men who have sex with men [22]. Although the authors refer to “possible interventions” in the appendix, these recommendations are not integrated into the discussion.

Lastly, “actionable” narratives specify strategies for dismantling racism based on the understanding that structural racism (e.g., policies, unwritten rules) produces racial/ethnic health inequities. To illustrate this level in Table 3, we provided an excerpt from a 2020 commentary in NEJM in which the authors advocate for explicitly anti-racist changes to the health care system [23].

These broad levels illustrate the range of narratives within medical journal articles within the anti-racism construct. Each broad level provides insight into the mechanisms by which racism narratives influence norms and spur action. Narratives that dismiss racism as a determinant of health (0 - impedes anti-racism) hinder anti-racism efforts. Narratives that describe how racism produces racial health inequities document differences at the person or societal levels (1 - weak anti-racism and 2 - moderate anti-racism). These narratives could potentially inform anti-racist strategies. Yet to dismantle systemic racism, narratives must advance strategic collaboration through concrete actions (3 - strong anti-racism). The broad levels, while comprehensive, parsimoniously span the anti-racism spectrum. Therefore, we concurrently developed granular modalities to capture further nuance and complexity of racism narratives.

### Granular modalities

We additionally devised 12 granular modalities to illustrate the multifaceted nuances of racism narratives. These labels indicate whether racism is explicitly named, the domain in which racism is being named, and the extent to which the excerpt contributes toward dismantling racist structures. To demonstrate the utility of the granular modalities, we highlight two excerpts that impede anti-racism (i.e., scored as 0 on the anti-racism spectrum) but vary in their method of dismissal. Table 3 presents example excerpts with both labels to illustrate the range of language used to explain differences in health outcomes by race.

In the following excerpt, the authors name “racism” as a potentially harmful byproduct of using race in “medical decision making”:

*The geneticist J. Craig Venter, on completion of mapping his own genome, famously asserted that “race is a social construct, not a scientific one.” If this is true, there is little reason to let the concept of race enter into medical decision making. Some physicians have expressed concern that “racial profiling” in medicine could cause harm by promoting stereotyping, bias, or even racism.* [24].

(Granular modality Minimizes racism as a mechanism)

Describing some physicians’ concerns regarding the clinical relevance of “race” reflects a race-evasive approach to racism; that is, if physicians do not acknowledge race in the exam room, the patient’s health is somehow isolated from their reality of living in a racist society. Of note, the term ‘racial profiling’ is used incorrectly here, as it implies discriminatory practices (unfair targeting of individuals based on their race, often leading to discrimination and unjust treatment). This narrative minimizes how structural racism, e.g., access to economic opportunities, quality education, and health care, produces poor health [2].

The following excerpt focuses on biological reasons for race-related differences in cancer survival:

*Many researchers and physicians have concluded that poorer survival of blacks relative to whites after a cancer diagnosis reflects fundamental differences in the biology of the host or the attendant cancer or both. We did not observe the impact of these putative biological differences consistently in cohorts of comparably treated black and white patients with cancer of similar stage once we took into account differences in underlying death rates. We cannot be sure if our findings in breast cancer, uterine cancer, and bladder cancer constitute exceptions to this conclusion or reflect residual differences in treatment and disease severity that could not be identified through our study.* [25].

(Granular Modality Attributes race-related difference in health to biomedical (natural) factors)

After adjusting for underlying death rates, the study did not find “biological differences” between Black and white patients at similar stages of cancer who received similar treatment. The authors acknowledge that such findings do not align with the dominant narrative of biological differences. Given the weight of other research that has assumed biological differences, the authors hesitate to explicitly dismiss biological differences and offer alternative explanations for their results. Whether such reluctance reflects the authors’ interpretations or a stance diluted through the peer-review process, the biological narrative is ultimately not refuted.

Neither of these excerpts advance anti-racism research for health equity, and so are assigned the same broad level. However, the precise way racism is discussed differs, signaling different granular modalities. The former advocates for keeping race out of medicine altogether whereas the latter suggests that a lack of race-related biological differences may be ‘exceptions’ or due to measurement error. This supports a harmful narrative of “biologic essentialism”, despite overwhelming evidence disputing biological or genetic differences based on race as a reason for racial health inequities [8].

Lastly, we examined the distribution of excerpts across both broad levels and granular modalities in a cross table (Table 4). The distribution of excerpts labeled with broad levels generally overlapped with those labeled with granular modalities. Coders agreed on which excerpts were clearly anti-racist. Excerpts categorized in the “actionable” broad level (3 - strong anti-racism) were consistently coded with anti-racist granular modalities (3 - strong anti-racism). Excerpts labeled as the “dismissal” broad level (0 - impedes anti-racism) were mostly labeled on the lower end for granular modalities (0 - impedes anti-racism).

**Table 4:** Coded excerpts by broad and granular labels

	Broad Level					
Granular Modality	Dismissal	Person-level	Societal	Actionable	Not enough info	Total
Dismisses racism as a mechanism; does not name racism	1					1
Minimizes racism as a mechanism	3					3
Documents race-related difference in health without naming a determinant; does not name racism	4	1				5
Attributes race-related difference in health to biomedical (natural) factors	5					5
Attributes race-related difference in health to interpersonal (aberrant) factors	4	2				6
Attributes race-related difference in health to social or institutional (environmental); does not name racism	5	1	1	1	1	9
Names racism among several possible determinants of health		4	3			7
Attributes race-related difference in health to social or institutional (environmental); names racism		5		2	1	8
Attributes race-related difference in health to social or institutional (environmental); recommends action			1			1
Describes structural (embedded into society) factors; does not name racism	1	1	5		2	9
Describes racism as structural (embedded into society); names racism		1	1	8		10
Describes racism as structural (embedded into society); recommends action		3	7	5	1	16
Grand Total	23	18	18	16	5	80

However, some excerpts were less explicit and yielded seemingly incongruent labels. For example, one excerpt was labeled the broad level of “dismissal” and the granular modality of “Describes structural (embedded into society) factors; does not name racism.” Such exceptions reveal the utility of the granular modalities and can shed light on the manner in which racism narratives can be expressed. For example, labeler perspective and context can influence coding, as revealed by the granular modalities (see Supplemental Table 4 for examples). Together, these results demonstrate that a coupling of broad levels and granular modalities facilitates nuanced examination of racism narratives in the medical literature.

## Discussion

In this qualitative study, we developed a construct of broad and granular racism narratives, developed from excerpts of articles in influential medical journals. We arranged these narratives on a spectrum from dismissal of racism as a cause of health inequities to strong anti-racism describing actionable strategies against structural racism.

Medical leadership, clinicians, and educators may benefit from this framework in several ways. First, the framework can serve as a guide to more critically evaluate how published medical research and education is consumed [26–28]. The racism narratives we identified have shaped our current body of literature on health inequities and likely influenced the attitudes, beliefs, and views of the medical field on the role of racism on health and healthcare systems. Becoming aware of the narratives identified in this framework will help health professionals, medical educators, and students re-evaluate medical knowledge. Second, when confirmed in future work, this framework can be adapted and implemented into anti-racist medical curricula. Third, for medical researchers, this framework can be a first step for incorporating anti-racist narratives on health inequities in medical research. Finally, this framework also provides insight for policymakers into how health systems can shift towards anti-racist narratives. We urge medical leadership, journal editors, and policymakers to critically consider how racism narratives are framed in novel research works and develop ways of generating and disseminating knowledge on racism and health inequities. To shift racism narratives, efforts from the larger medical eco-system are crucial.

### Relation to previous work

Our work advances an understanding of which racism narratives currently exist and how to measure them. Building upon prior quantifications of racism in medical literature, [5, 7, 29, 30] we qualitatively documented text sourced from leading medical journals to illustrate a range of racism narratives. Dominant narratives that dismiss racism or focus on downstream risk factors (e.g., person-level behaviors) render invisible the equity-focused narratives about upstream influences such as historical and contemporary systems of power. This aligns with recent studies that underscore the importance of language for health equity [7, 30]. We add to this literature an empirically supported theorization of racism narratives.

### Strengths and limitations

We used a novel approach to examine narratives in medical literature. We demonstrated qualitative variation in the understanding of racism using published literature as evidence. Furthermore, our independent coding process enabled two ordering schemes to develop, which prompted nuanced conversations regarding context. Relatedly, the granular modalities depict the complexity of racial health equity research and the need for researchers to cultivate theoretical sensitivity to racialization.

Our exploratory approach also had its limitations. Simultaneous coding of broad and granular labels illustrated the difficulty in categorizing excerpts due to the biases inherent in manual coding. Because we acknowledge the social construction of knowledge, our goal was not to reach perfect agreement, but rather to recognize biases and explicitly identify how they shape our interpretation. Measurement theory serves as a paradigm for which these biases–reframed as annotator perspective–can be explicitly modeled, more fully accounting for how nuanced excerpts are labeled in the construct^15^.

Relatedly, racism manifests differently for different racialized groups. Precisely identifying the specific ways that racially marginalized groups are stereotyped, stigmatized, and disadvantaged could refine the levels of racism narratives. Additionally, several excerpts recommended structural changes without explicitly naming racism whereas other excerpts recommended actions that did not address structural racism. How we should evaluate these ‘mixed’ articles remains a topic for further deliberation.

Further, our study focuses on papers published in high-impact journals that influence the medical field: the New England Journal of Medicine (NEJM), The Lancet, the Journal of the American Medical Association (JAMA), and the British Medical Journal (BMJ). Thus, these articles more often, though not exclusively, describe the situation in European countries and settler colonies (US, Canada, Australia, New Zealand). Further other health fields, such as public health, are less represented, and our narratives were constructed using articles spanning a broad time period. Finally, our study was limited to articles that mentioned the word “racism” in the body of the text. Narratives in articles that use related concepts to describe manifestations of racism (e.g., discrimination, bias, etc.) were out of scope. In future work, we aim to include other health journals, examine how narratives change over time, and expand our analysis to other articles that discuss racial health inequities.

### Future research

Future research should build upon the developed construct of racism narratives to increase its utility as a methodological tool. In Rasch measurement theory, a construct is operationalized through a data collection instrument (e.g. exam, survey, or annotation guidelines), and collected responses can be transformed into a numerical scale capable of measuring the underlying phenomenon. Measurement scales, heavily used in fields such as education, [31] have recently been leveraged in natural language processing, such as hate speech research [14, 16]. Further refinement of this construct could pave the way to tools that support measurement of racism narratives in the medical literature at scale and over time.

## Conclusion

We constructed a framework of racism narratives in medical literature, ranging from impeding anti-racism to strong anti-racism. Our analysis depicted dismissal, person-level, societal, and actionable narratives in influential medical journals. More research must disrupt race-evasive narratives and expose the harms of racism on health. We encourage clinicians, educators, and policymakers to acknowledge that these narratives exist in influential medical literature, to more critically evaluate how racial health equity research is consumed, and to create novel research and education paradigms that disrupt current norms in medicine. Race-conscious, action-oriented narratives are necessary to ensure health equity and anti-racist transformations across the medical ecosystem.

### Electronic supplementary material

Below is the link to the electronic supplementary material.

Supplementary Material 1

## Data Availability

Data will be made available on request.

## Declarations

### Disclaimer

The ideas in this article are those of the authors and do not necessarily represent the policy of the American Medical Association.
