# Correction: The stories about racism and health: the development of a framework for racism narratives in medical literature using a computational grounded theory approach

**DOI:** 10.1186/s12939-024-02095-6

**Published:** 2024-03-06

**Authors:** Caroline A. Figueroa, Erin Manalo-Pedro, Swetha Pola, Sajia Darwish, Pratik Sachdeva, Christian Guerrero, Claudia von Vacano, Maithili Jha, Fernando De Maio, Chris J. Kennedy

**Affiliations:** 1 University of California Berkeley, Berkeley, USA; 2 Department of Technology, Policy, and Management, Delft University of Technology, Policy & Management Room B3.230, Building 31, Jaffalaan 5, 2628 BX Delft, The Netherlands; 3 Fielding School of Public Health, University of California Los Angeles, Los Angeles, USA; 4 School of Public Health, University of California Berkeley, Berkeley, USA; 5 American Medical Association, Chicago, USA; 6 Department of Sociology, DePaul University, Chicago, USA; 7 Department of Psychiatry, Harvard Medical School, Boston, USA; 8 Center for Precision Psychiatry, Massachusetts General Hospital, Boston, USA

**International Journal for Equity in Health (2023) 22:265**

10.1186/s12939-023-02077-0

After publication of this article [1], the authors reported that the disclaimer statement in the backmatter was missing and should have read ‘Disclaimer: The ideas in this article are those of the authors and do not necessarily represent policy of the American Medical Association.’

The original article [1] has been corrected.
