# Supplementary material for: The stories about racism and health: the development of a framework for racism narratives in medical literature using a computational grounded theory approach
Source: Int J Equity Health. 2023 Dec 21;22:265. doi: 10.1186/s12939-023-02077-0 (PMC10734166; doi:10.1186/s12939-023-02077-0)
Supplement: Supplementary file 1 — Supplementary Material 1 [file 12939_2023_2077_MOESM1_ESM.docx]

Supplementary material Figueroa et al.

## **Background on literature review**

We conducted multiple searches between August 2020 and March 2021 in Pubmed and Google Scholar using a combination of keywords related to racism (e.g. “racism”, “racist”, “racial”), medical literature (e.g. “health”, “medicine”, “medical journals”, and narratives (e.g. “narratives”, “discourse”, “concepts”, “conceptualization”, “mentioning”). We searched for systematic reviews, meta-analyses, and narrative reviews. Since the search retrieved fewer review articles than expected (we aimed for +/- 20 articles), we also included medical commentaries, articles from the education field, and recommendations from experts. These articles did not overlap with the articles used for the coding of excerpts (paragraphs from medical articles).  Based on the review, we developed four broad categories of narratives

## Results

From the literature search we identified 23 articles on publishing about racism in scientific health-related literature: 7 systematic reviews, 6 narrative reviews, and 3 commentaries within medicine, public health, nursing and medical education. The articles, a summary of their content, and our reasons for including them, are shown in the supplementary Table 1.

**Racism narrative lessons learned**

Below we describe the most important lessons learned from our narrative review:

- Racism as a cause of health inequities is infrequently discussed in medical research, and when discussed, is not a focal point of the paper.
- The analysis of racism in relation to health inequities focuses mainly on personal experiences (perceived discrimination), genetics or behavior (also referred to as ‘victim blaming’), and physicians’ implicit attitudes as opposed to structural inequities. Thus, we consider narratives that focus on race as biology and personal experiences, rather than racism as a system to be dominant. More critical race conscious narratives that explicitly name structural racism as a distal factor may be more rare.
- In addition, we consider prevalent narratives as those that omit the political origins of proximal social/environmental factors, whereas more critical race conscious narratives regard these social contexts as manifestations of racism.
- If narratives mention solutions, they are more often directed at individual behavior change, interpersonal attitudes, or unspecified forms of racism, whereas more critical race conscious narratives would argue for more structural change.

**Table S1. Summary of articles**

| **Article Title** | **Year** | **Aim** | **Method** | **Finding** |
| --- | --- | --- | --- | --- |
| **Systematic Reviews** | | | | |
| 1. Naming Institutionalized Racism in the Public Health Literature: A Systematic Literature Review [(Hardeman et al. 2018)](https://paperpile.com/c/RiGhHm/M0eq)* | 2018 | The goal of this analysis was to review the contemporary peer-reviewed public health literature from 2002-2015 to determine whether the concept of institutionalized racism was named (ie, explicitly mentioned) and whether it was a core concept in the article. | Systematic literature review methodology to find articles from the top 50 highest-impact journals in each of 6 categories (249 journals in total) that most closely represented the public health field, were published during 2002-2015, were US focused and mentioned terms relating to institutionalized racism in their titles or abstracts. | Only 25 articles named institutionalized racism in the title or abstract among all articles published in the public health literature during 2002-2015 in the 50 highest-impact journals and 6 categories representing the public health field in the United States. Institutionalized racism was a core concept in 16 of the 25 articles. |
| 2. Public Health’s Approach to Systemic Racism: a Systematic Literature Review [(Castle et al. 2019)](https://paperpile.com/c/RiGhHm/yCmU)* | 2019 | The purpose of this systematic literature review was to analyze the extent to which public health currently addresses systemic racism in the published literature. | Utilizing the PRISMA guidelines, this review examines three widely used databases to examine published literature covering the topic as well as implications for future research and practice. | Included 85 papers. Across numerous articles, the terms racism and systemic racism are largely absent. Discrimination, stigma, and bias were used to describe (systemic) racism. Many (excluded) articles focused on the effects of racism on behavior. Few implications for changes at the systemic level. |
| 3. Racial Inequality in Psychological Research: Trends of the Past and Recommendations for the Future [(Roberts et al. 2020)](https://paperpile.com/c/RiGhHm/1wUS) | 2020 | Authors examined how often psychological research acknowledges race and whether people who edit, write, and participate in the research are systematically connected. | Queried more than 26,000 empirical articles published between 1974 and 2018 in top-tier cognitive, developmental, and social psychology journals | Psychological publications that highlight race have been rare. Increased in developmental and social psychology, they have remained virtually nonexistent in cognitive psychology. Most publications have been edited by White editors, publications that highlight race have been written by white authors. Few authors of color. |
| 4. Discourse of race and racism in nursing: An integrative review of literature. [(Iheduru-Anderson, Shingles, and Akanegbu 2021)](https://paperpile.com/c/RiGhHm/FpPQ) | 2016 | Determine whether racism and institutionalized racism are explicitly named in the titles and abstracts of peer-reviewed publications on nursing education, leadership, and the nursing profession, and to explore the depth of discussion of racialized concepts in peer-reviewed nursing literature. | 23 studies mentioned racism in abstract or title. An integrative review approach was used to review 23 studies published in nursing journals published from 2008 to 2020. | Mostly qualitative studies about experiences of nurses. Some of the studies linked racism and the racialized policies to the experiences of the nurses or students in the study. |
| 5. Words Matter: An Integrative Review of Institutionalized Racism in Nursing Literature [(Thurman, Johnson, and Sumpter 2019)](https://paperpile.com/c/RiGhHm/MDt7) | 2019 | Identify how and to what extent peer-reviewed nursing literature and professional nursing organizations have explicitly addressed institutionalized racism | A systematic search of relevant nursing literature published since 2008. 29 journal articles that focused on black Americans' experience of institutionalized racism in health and health care. | The 29 articles included in this review were  published in 15 journals, fewer than 10% of  the nursing journals searched. |
| 6. Racism as a Determinant of Health: A Systematic Review and Meta-Analysis | 2015 | meta-analysis on the relationship between reported racism and mental and physical health outcomes | Looked at self-reported measures (experienced or internalized) and exposure measures (including discrimination, maltreatment, prejudice, stereotypes, aggression).  Data from 293 studies reported in 333 articles published between 1983 and 2013, and conducted predominantly in the U.S. | Racism was associated with poorer mental, general and physical health.  “One of the key challenges in the study of racism and health is the profusion of exposure measurements currently utilized by researchers”. |
| 7. Racism and health service utilisation: A systematic review and meta-analysis [(Ben et al. 2017)](https://paperpile.com/c/RiGhHm/h3CN) | 2017 | Systematic review and meta-analysis of self-reported racism and healthcare service utilization | Systematic review of 83 papers reporting 70 studies. Studies included 250,850 participants and were conducted pre- dominately in the U.S. The meta-analysis included 59 papers reporting 52 studies. | Racism was associated with more negative patient experiences of health services, but not most indicators of health service use. Racism was associated with delay of care and lack of adherence. |
| **Narrative reviews** | | | | |
| 8. Structural racism and health inequities in the USA: evidence and interventions [(Bailey et al. 2017)](https://paperpile.com/c/RiGhHm/RgjZ) | 2017 | Examine what constitutes structural racism, explore evidence of how it harms health, and provide examples of interventions that can reduce its impact. | Conceptual report. | Most studies have focused on interpersonal racial/ethnic discrimination, less emphasis on health effects of structural racism. |
| 9. Racial inequalities in health: Framing future research [(Hicken et al. 2018)](https://paperpile.com/c/RiGhHm/SEKA) | 2018 | Introducing a special collection on health inequities. The paper discusses the definitions of cultural and structural racism and provides recommendations for future research on racial health inequalities. | Report/review | Provides a call to action for moving toward a “reconstruction of knowledge” about the root causes of inequalities. |
| 10. On Racism: A New Standard For Publishing On Racial Health Inequities [(Boyd, Lindo, and Weeks 2020)](https://paperpile.com/c/RiGhHm/1oCv)* | 2020 | Guidelines for research and journals in addressing race | Review/opinion | A quick search of the Health Affairs website revealed only 114 pieces include the word racism in the 39-year history of the journal. Most articles in the medical canon that use the term “race” do not additionally use the terms “structural racism” or “systemic racism.” Article provides recommendation for authors on publishing. |
| 11. Defining, conceptualizing and characterizing racism in health research [(Paradies 2006)](https://paperpile.com/c/RiGhHm/ZODY) | 2006 | Examining the study of racism and health, using three different levels. | Uses a previous systematic review as a guide, which identified 138 published studies on racism as a social determinant of health, of which 65% were published between 2000 and 2004 | Recommendations: To conceptualize racism as a social determinant of health, we need to: define and theorize racism itself, clarify how racism may relate to health and characterize racism as an exposure that can be operationalized and measured. Future research needs to examine privileges of racism (whiteness studies) power analyses, and subjective vs. objective racism. |
| 12. The Conceptualization of Everyday Racism in Research on the Mental and Physical Health of Ethnic and Racial Groups: a Systematic Review [(Bourabain and Verhaeghe 2020)](https://paperpile.com/c/RiGhHm/9pFF)* | 2020 | How is the concept of everyday racism and discrimination defined in health studies? | A systematic review perusing 106 papers of studies investigating the relationship of everyday racism/discrimination and the physical and mental health of ethnic and/or racial groups. | Racism was only defined in 7% of the papers and in different ways varying from individual to institutional racism. 86% of the papers measured everyday racism/discrimination through the Everyday Discrimination Scale. This influenced the way in which everyday discrimination was defined, taking on a more individual perspective paying less attention to the micro-macro link of everyday racism. |
| 13. How Social Welfare and Health Professionals Understand “Race,” Racism, and Whiteness: A Social Justice Approach to Grounded Theory [(Vanidestine and Aparicio 2019)](https://paperpile.com/c/RiGhHm/cvhT) | 2020 | Semi-structured interviews with physicians and Social Workers | Social welfare and health professionals’ understand race, racism, and whiteness: (1) self-defined skin color; (2) (pre)judgments and discrimination; (3) privilege and power; and (4) conceptual conflation and unfamiliarity. | More emphasis during training and in workplace settings on concepts such as power, inequities, and structural racism may significantly improve the efficacy and scope of interventions. |
| 14. Medicine’s Privileged Gatekeepers: Producing Harmful Ignorance About Racism And Health. Health affairs Blog.  [(Krieger et al. 2021)](https://paperpile.com/c/RiGhHm/88ZX)***** | 2021 | Literature search papers including the word “racism” published between January 1, 1990 and December 31, 2020. Journals were: the [New England Journal of Medicine](https://www.nejm.org/) (NEJM), [The Lancet](https://www.thelancet.com/), [JAMA](https://jamanetwork.com/), and the [British Medical Journal](https://www.bmj.com/) (BMJ). comparison: the [American Journal of Public Health](https://ajph.aphapublications.org/) (AJPH), and we additionally examined the [Annual Review](https://www.annualreviews.org/). | Blog/SR | All four medical journals and AJPH increases in articles with word “racism” in 2020 (but low before this). The four medical journals lower numbers of papers including the word “racism. In the 4 journals only +- 10% were empirical studies. Of these 64 medical journal articles, only 10 (15.6 percent) included any substantive [discussion of theory](https://oxford.universitypressscholarship.com/view/10.1093/acprof:oso/9780195383874.001.0001/acprof-9780195383874) guiding the research. 18 (28.1 percent) tested a hypothesis. “racism” mainly used in discussion section, not focal point of the paper. Fewer than 10 percent included any measures of structural, institutional, interpersonal, or internalized racism. |
| 15. Is Social Work Still Racist? A Content Analysis of Recent Literature [(Corley and Young 2018)](https://paperpile.com/c/RiGhHm/bj25) | 2018 | Content analysis of articles on Asian Pacific Islander (API) Americans, African Americans, Latinx or Hispanic Americans, and Native or Indigenous Americans in four major social work journals published between 2005 and 2015. | Re-do of a former content analysis McMahon and Allen-Meares (1992). Authors selected three of the four original journals for review: Social Work, Child Welfare, and Social Service Review. | Majority of articles (65.85 percent) focused on individual interventions, i.e. cultural competence of social workers. The profession is still failing to address institutional racism. |
| **Commentaries** | | | | |
| 16. Responsibility of Medical Journals in Addressing Racism in Health Care [(Ogedegbe 2020)](https://paperpile.com/c/RiGhHm/BXLg) | 2020 | Reassess the role of medical journals in addressing the health effects of systemic racism given the recent protests in the US after the death of George Floyd. | Opinion. | The author provides recommendations for medical journals. 1. Focus attention on the issues of structural racism in health care institutions and large hospital chains. Publish papers on this topic, establish panels at medical conferences and publish proceedings at special issues. Publish papers on the effect of structural racism on health and on the lack of progress of increasing the number of black men in medicine.  2. Increase the number of black, Hispanic, and indigenous scholars as editors and editorial boards of major medical journals. 3. Instill hope. E.g. publish case studies, interventions, and success stories targeting structural racism in the United States. |
| 17. How Structural Racism Works — Racist Policies as a Root Cause of U.S. Racial Health Inequities.  [(Bailey, Feldman, and Bassett 2021)](https://paperpile.com/c/RiGhHm/NoIy) | 2020 | Explain how structural racism leads to health inequities, and what our (researchers, doctors, journals) role is in dismantling structural racism. | Opinion/editorial. | There is no “official” definition of structural racism, but all definitions make clear that racism is not simply the result of private prejudices, but produced by laws, rules, and practices, sanctioned, and implemented by various levels of government, and embedded in the economic system as well as in cultural and societal norms.  Recommendations: Funders, editors, and reviewers — racism and inequities in social determinants of health are topics as valid for research as biologic markers.  Improve race and ethnicity data and the measurement of structural racism.  Recognize harms arising from the uncritical use of racial categories, e.g. racial differences are genetic. Measure the success of interventions in narrowing inequitable gaps in health.  Address shortage of black students and researchers. |
| 18. How to Measure Racism in Academic Health Centers. [(Adkins-Jackson, Legha, and Jones 2021)](https://paperpile.com/c/RiGhHm/gzzC) | 2021 | Proposing a measure of institutional racism in Academic Health Centers. | Opinion. Previous measures of institutional racism focus only on individuals’ perceptions of racism on the assumption that racism must be encountered by an individual in order for institutional policies to be racist. | Scores on measures 3 levels: individual (perceived racism, clinician racism), intra-organizational (how clinicians are trained), extra-organizational (policies, services, distribution, etc.), yields a composite score of institutional racism. This can inform antiracist strategic planning and decision making . |
| 19. The public health critical race methodology: Praxis for antiracism research [(Ford and Airhihenbuwa 2010)](https://paperpile.com/c/RiGhHm/GMup) | 2010 | Introduces framework for applying Critical Race Theory to health equity research | Opinion/review | Four focuses:   1. contemporary racial relations 2. knowledge production 3. conceptualization and measurement 4. action |
| 20. Toward The Science and Practice of anti-racism: Launching a national campaign against racism [(Jones 2018)](https://paperpile.com/c/RiGhHm/pOWl) | 2018 | Setting the agenda for the National Campaign Against Racism | Editorial | 1. Name racism 2. Ask “how is racism operating here?” 3. Organize and strategize to act |
| **Other** | | | | |
| 21. Race without Racism: How Higher Education Researchers Minimize Racist Institutional Norms [(Harper 2012)](https://paperpile.com/c/RiGhHm/PRPI)* | 2012 | How do higher education scholars discuss and make sense of race-related findings that emerge in their studies? Systematic review. | Systematic review of articles in 7 peer-reviewed academic journals of education research. | 255 articles identified.  Authors discussing racism use words such as: “perhaps,” “may,” “might,” “possibly,” “could be,” and “presumably.” Rarely were racism and racist institutional norms explicitly named among the range of plausible reasons for racial differences.  Many semantic alternatives to “racism” and “racist”:  Only 5 studies used critical race theory. |
| 22. Content Analysis of Textbooks via Natural Language Processing: Findings on Gender, Race, and Ethnicity in Texas U.S. History Textbooks [(Lucy et al. 2020)](https://paperpile.com/c/RiGhHm/3HFv) | 2020 | Apply NLP methods to examine depictions of historically marginalized groups in textbooks | Content analysis. Analyzed: How much space is allocated to different groups? How are they described? What are prominent topics and how are they related to different groups?  Used methods such as Wordnet, National research council Lexicon, and topic modeling. | Women are mentioned in the contexts of marriage, home, and work. Black people are involved in actions with low agency and power. Books focus more on political history than social history, and discussions of minority ethnicities center on their relationships with White people. |
| 23. Discourse and the denial of racism [(van Dijk 1992)](https://paperpile.com/c/RiGhHm/6mkz) | 1992 | Examines the prominent role of the denial of racism, especially among the elites. | Analysis of conversations, press reports and parliamentary debates | Among these forms of denial are disclaimers, mitigation (downtoning, minimizing or using euphemisms), excuses, blaming the victim, reversal and other moves of defence, face-keeping and positive self-presentation in negative discourse about minorities, immigrants and (other) anti-racists. |

*key articles

**Table S2.** Excerpts clustered by journal

|  | *Broad Category* | | | |  |  |
| --- | --- | --- | --- | --- | --- | --- |
| *Journal* | Dismissal | Person-level | Societal | Actionable | Not enough info | Grand Total |
| BMJ | 5 | 5 | 1 | 2 | 1 | 14 |
| JAMA | 9 | 3 | 4 | 1 | 2 | 19 |
| NEJM | 3 | 4 | 3 | 8 | 1 | 19 |
| The Lancet | 6 | 6 | 10 | 5 | 1 | 28 |
| **Grand Total** | **23** | **18** | **18** | **16** | **5** | **80** |

**Table S3.** Excerpts clustered by year

|  | *Broad Category* | | | |  |  |
| --- | --- | --- | --- | --- | --- | --- |
| *Year* | Dismissal | Person-level | Societal | Actionable | Not enough info | Grand Total |
| 1960 - 1969 | 1 |  |  | 1 |  | 2 |
| 1970 - 1979 | 2 | 1 | 1 |  |  | 4 |
| 1980 - 1989 | 3 | 1 |  |  |  | 4 |
| 1990 - 1999 |  | 2 |  |  |  | 2 |
| 2000 - 2009 | 5 | 6 | 9 | 4 | 2 | 26 |
| 2010 - 2019 | 12 | 6 | 5 | 1 | 2 | 26 |
| 2020 - 2029 |  | 2 | 3 | 10 | 1 | 16 |
| **Grand Total** | **23** | **18** | **18** | **16** | **5** | **80** |

**Table S4.** Examples of nuances captured by granular modalities

| Example Excerpt | Broad Level with Explanation | Granular Modality with Explanation |
| --- | --- | --- |
| *Doing so starts with ensuring access to quality health care for all women. Despite the gains under the Affordable Care Act, nearly 1 in 7 US women of childbearing age remain uninsured. Proposed Medicaid waivers in some states to restrict eligibility, enrollment, and benefits; increasing health care premiums and deregulation of Affordable Care Act health plans; proposed religious and moral exemptions for contraceptive coverage; and defunding of Planned Parenthood could further reduce access to primary and preventive health services, preconception and interconception care, family planning, and other services vital to protecting women’s health.*[*^30^*](https://paperpile.com/c/vRbxmq/Nq3d) | Dismissal  This excerpt did not explicitly mention racism nor women of color. | 10 - Describes structural (embedded into society) factors; does not name racism  The excerpt’s author framed “access to quality health care for all women” through federal policy as a structural factor.  Label was influenced by the coder’s awareness of maternal mortality’s disproportionate burden among Black women. |
| *Furthermore, “Cultural identity depends not only on access to culture and heritage but also on opportunities for cultural expression and cultural endorsement within society’s institutions. Identity [as understood within an Indigenous context] is to a large extent a collective experience.” Moreover, Brant has opined that in view of these losses, Indigenous peoples can often be overtaken by repressed hostility that comes from cultural prohibitions against showing angry behaviour. This hostility gives rise to explosiveness under the influence of alcohol, and to a high frequency of grief reactions.* | Dismissal  Racism is not explicitly mentioned in this excerpt. | 06 - Attributes race-related difference in health to social or institutional (environmental); does not name racism  The excerpt describes the sequelae of urbanization as creating barriers for Indigenous peoples to access the protective factors of “cultural expression.”  Label was influenced by the coder’s knowledge of colonialism as a determinant of health. |
| *The study results raise the question of whether intensive, individual-level interventions are sufficiently broad and robust enough to improve HIV outcomes among populations currently not benefitting from treatment. Most participants were low-income persons of color who may experience negative socio cultural factors such as poverty, racism, unstable housing, HIV-related stigma, and high rates of incarceration. Systemic and structural barriers to care may be difficult to overcome with an individual-level behavioral intervention, even an intensive one; for example, this study found that black participants (compared with white participants) and participants from southern sites were less likely to be virally suppressed. ... other approaches are needed to improve HIV outcomes in this vulnerable population.* | Societal  This excerpt names “sociocultural factors” and “systemic and structural barriers.” | 12 - Describes racism as structural (embedded into society); recommends action  The author argues that “other approaches” are needed to address the structural factors that disproportionately affected the Black participants in the study. By naming the inadequacy of intensive, individual-level interventions, the author encourages taking action at the societal level.  Label was influenced by the coder’s understanding of the limitations of individual-level interventions. |

## **References**

[Adkins-Jackson, Paris B., Rupinder K. Legha, and Kyle A. Jones. 2021. “How to Measure Racism in Academic Health Centers.” *AMA Journal of Ethics* 23 (2): E140–45.](http://paperpile.com/b/RiGhHm/gzzC)

[Bailey, Zinzi D., Justin M. Feldman, and Mary T. Bassett. 2021. “How Structural Racism Works — Racist Policies as a Root Cause of U.S. Racial Health Inequities.” *The New England Journal of Medicine* 384 (8): 768–73.](http://paperpile.com/b/RiGhHm/NoIy)

[Bailey, Zinzi D., Nancy Krieger, Madina Agénor, Jasmine Graves, Natalia Linos, and Mary T. Bassett. 2017. “Structural Racism and Health Inequities in the USA: Evidence and Interventions.” *The Lancet* 389 (10077): 1453–63.](http://paperpile.com/b/RiGhHm/RgjZ)

[Ben, Jehonathan, Donna Cormack, Ricci Harris, and Yin Paradies. 2017. “Racism and Health Service Utilisation: A Systematic Review and Meta-Analysis.” *PloS One* 12 (12): e0189900.](http://paperpile.com/b/RiGhHm/h3CN)

[Bourabain, Dounia, and Pieter-Paul Verhaeghe. 2020. “The Conceptualization of Everyday Racism in Research on the Mental and Physical Health of Ethnic and Racial Groups: A Systematic Review.” *Journal of Racial and Ethnic Health Disparities*, August. https://doi.org/](http://paperpile.com/b/RiGhHm/9pFF)[10.1007/s40615-020-00824-5](http://dx.doi.org/10.1007/s40615-020-00824-5)[.](http://paperpile.com/b/RiGhHm/9pFF)

[Boyd, R. W., E. G. Lindo, and L. D. Weeks. 2020. “On Racism: A New Standard For Publishing On Racial Health Inequities.” healthaffairs.org. 2020.](http://paperpile.com/b/RiGhHm/1oCv) <https://www.healthaffairs.org/do/10.1377/hblog20200630.939347/full/?utm_medium=social&utm_sour>[.](http://paperpile.com/b/RiGhHm/1oCv)

[Castle, Billie, Monica Wendel, Jelani Kerr, Derrick Brooms, and Aaron Rollins. 2019. “Public Health’s Approach to Systemic Racism: A Systematic Literature Review.” *Journal of Racial and Ethnic Health Disparities* 6 (1): 27–36.](http://paperpile.com/b/RiGhHm/yCmU)

[Corley, Nicole A., and Stephen M. Young. 2018. “Is Social Work Still Racist? A Content Analysis of Recent Literature.” *The Social Worker* 63 (4): 317–26.](http://paperpile.com/b/RiGhHm/bj25)

[Dijk, Teun A. van. 1992. “Discourse and the Denial of Racism.” *Discourse & Society* 3 (1): 87–118.](http://paperpile.com/b/RiGhHm/6mkz)

[Ford, Chandra L., and Collins O. Airhihenbuwa. 2010. “The Public Health Critical Race Methodology: Praxis for Antiracism Research.” *Social Science & Medicine* 71 (8): 1390–98.](http://paperpile.com/b/RiGhHm/GMup)

[Hardeman, Rachel R., Katy A. Murphy, J ’mag Karbeah, and Katy Backes Kozhimannil. 2018. “Naming Institutionalized Racism in the Public Health Literature: A Systematic Literature Review.” *Public Health Reports* 133 (3): 240–49.](http://paperpile.com/b/RiGhHm/M0eq)

[Harper, Shaun R. 2012. “Race without Racism: How Higher Education Researchers Minimize Racist Institutional Norms.” *The Review of Higher Education* 36 (1): 9–29.](http://paperpile.com/b/RiGhHm/PRPI)

[Hicken, Margaret T., Nicole Kravitz-Wirtz, Myles Durkee, and James S. Jackson. 2018. “Racial Inequalities in Health: Framing Future Research.” *Social Science & Medicine* 199 (February): 11–18.](http://paperpile.com/b/RiGhHm/SEKA)

[Iheduru-Anderson, Kechi, René Revis Shingles, and Christiana Akanegbu. 2021. “Discourse of Race and Racism in Nursing: An Integrative Review of Literature.” *Public Health Nursing* 38 (1): 115–30.](http://paperpile.com/b/RiGhHm/FpPQ)

[Jones, Camara Phyllis. 2018. “Toward the Science and Practice of Anti-Racism: Launching a National Campaign Against Racism.” *Ethnicity & Disease* 28 (Suppl 1): 231–34.](http://paperpile.com/b/RiGhHm/pOWl)

[Krieger, Nancy, Rhea W. Boyd, Fernando De Maio, and Aletha Maybank. 2021. “Medicine’s Privileged Gatekeepers: Producing Harmful Ignorance About Racism And Health.” Health Affairs Blog. April 20, 2021.](http://paperpile.com/b/RiGhHm/88ZX) <https://www.healthaffairs.org/do/10.1377/hblog20210415.305480/full/>[.](http://paperpile.com/b/RiGhHm/88ZX)

[Lucy, Li, Dorottya Demszky, Patricia Bromley, and Dan Jurafsky. 2020. “Content Analysis of Textbooks via Natural Language Processing: Findings on Gender, Race, and Ethnicity in Texas U.S. History Textbooks.” *AERA Open* 6 (3): 2332858420940312.](http://paperpile.com/b/RiGhHm/3HFv)

[Ogedegbe, Gbenga. 2020. “Responsibility of Medical Journals in Addressing Racism in Health Care.” *JAMA Network Open* 3 (8): e2016531.](http://paperpile.com/b/RiGhHm/BXLg)

[Paradies, Yin C. 2006. “Defining, Conceptualizing and Characterizing Racism in Health Research.” *Critical Public Health* 16 (2): 143–57.](http://paperpile.com/b/RiGhHm/ZODY)

[Roberts, Steven O., Carmelle Bareket-Shavit, Forrest A. Dollins, Peter D. Goldie, and Elizabeth Mortenson. 2020. “Racial Inequality in Psychological Research: Trends of the Past and Recommendations for the Future.” *Perspectives on Psychological Science: A Journal of the Association for Psychological Science* 15 (6): 1295–1309.](http://paperpile.com/b/RiGhHm/1wUS)

[Thurman, Whitney A., Karen E. Johnson, and Danica F. Sumpter. 2019. “Words Matter: An Integrative Review of Institutionalized Racism in Nursing Literature.” *ANS. Advances in Nursing Science* 42 (2): 89–108.](http://paperpile.com/b/RiGhHm/MDt7)

[Vanidestine, Todd, and Elizabeth M. Aparicio. 2019. “How Social Welfare and Health Professionals Understand ‘Race,’ Racism, and Whiteness: A Social Justice Approach to Grounded Theory.” *Social Work in Public Health* 34 (5): 430–43.](http://paperpile.com/b/RiGhHm/cvhT)
